# Long noncoding RNA lnc-RI is a new regulator of mitosis via targeting miRNA-210-3p to release PLK1 mRNA activity

**DOI:** 10.1038/srep25385

**Published:** 2016-05-10

**Authors:** Zhi-Dong Wang, Li-Ping Shen, Cheng Chang, Xue-Qing Zhang, Zhong-Min Chen, Lin Li, Hong Chen, Ping-Kun Zhou

**Affiliations:** 1 Department of Radiation Toxicology and Oncology, Beijing Key Laboratory for Radiobiology, Beijing Institute of Radiation Medicine, Beijing, 100850, P. R. China; 2 Institute for Environmental Medicine and Radiation Hygiene, College of Public Health, University of South China, Hengyang, Hunan Province, 421000, P.R. China

## Abstract

Increasing evidence indicates that lncRNAs play critical roles in various biological processes, but many have not been functionally characterized. Here, we report a novel radiation-inducible lncRNA, namely lnc-RI which is essential for cell survival and appropriate mitotic progression. Our data indicated that knockdown of lnc-RI resulted in spindle abnormalities and mitotic arrest simultaneously with sharply decreased mRNA and protein expression of PLK1, a key regulator of mitosis. Our data demonstrated that PLK1 is a key downstream mediator of lnc-RI in regulating mitosis, whereby lnc-RI competitively bound to the negative PLK1 regulating miRNA, miRNA-210-p3. Taken together, we have identified lnc-RI as a new regulator of mitosis which acts by releasing PLK1 mRNA activity via competition for binding to miRNA-210-3p.

The mammalian genome transcribes numerous long noncoding RNAs (lncRNAs) but only a few of them have been characterized^1^. Increasing evidence indicates that lncRNAs play crucial roles in diverse biological and disease processes including modulation of apoptosis, carcinogenesis, immune responses, muscle differentiation, and neuronal development^2^,^3^,^4^,^5^,^6^,^7^,^8^,^9^. LncRNAs regulate targets through different mechanistic models. Some lncRNAs interact with heteronuclearribonucleo proteins or chromatin modification complexes in the nucleus^2^,^3^,^4^,^5^. Some lncRNAs affect mRNA stability in cytoplasm as competing endogenous RNAs (ceRNAs)^6^,^7^,^8^. A recent report has shown that other lncRNAs can bind directly to target proteins and promote protein phosphorylation^9^. However, there is very little information regarding the lncRNAs involved in the regulation of spindle formation and mitotic progression.

The mitotic process of mammalian cells is precisely controlled by a set of regulators which includes cyclins, cyclin-dependent kinases (CDKs), and CDK inhibitors. Polo-Like Kinase 1 (PLK1) is a key regulator of progression through mitosis from entry, spindle formation, cytokinesis to mitotic exit. In mammalian cells, dysregulation of PLK1 can result in the formation of aberrant spindles and mitotic arrest^10^. Mutations in PLK1 result in the formation of aberrant mitotic spindles and disrupt centrosome organization^11^,^12^,^13^. In U2OS cells, knockdown of PLK1 using siRNA induces mitotic arrest and aberrant spindle formation. In addition, reduction of PLK1 expression level or activity results in a decrease in cell growth and induces apoptosis in certain cancer cells^14^,^15^,^16^. An appropriate expression level of PLK1 is essential for the maintenance of genomic stability during mitosis. However, overexpression of PLK1 has been frequently detected in tumors, and PLK1 overexpression was reported to associate with chromosomal instability, DNA aneuploidy, and centrosome amplification^17^,^18^,^19^. It is likely that dysregulated PLK1 plays an active role in carcinogenic transformation and tumor growth^20^,^21^. In recent years, PLK1 has emerged as an attractive therapeutic target in oncology. Several PLK1 inhibitors have been developed showing encouraging results in early-phase clinical trials^22^,^23^.

Lnc-RI (lncRNA Radiation Induced) was originally identified as one of several lncRNAs which could be upregulated by ionizing radiation in our lab^24^. Up until now, its function has remained unclear. In this study, we demonstrated that lnc-RI is essential for cell survival and is involved in the regulation of proper spindle formation and mitotic progression by regulating PLK1 expression. We further found that both lnc-RI and PLK1 are targets of miRNA-210-3p, and lnc-RI regulates PLK1 mRNA stability by competing with the PLK1 mRNA 3′UTR for binding to miRNA-210-3p.

## Results

### Lnc-RI is essential for cell survival

**Figure 1:**
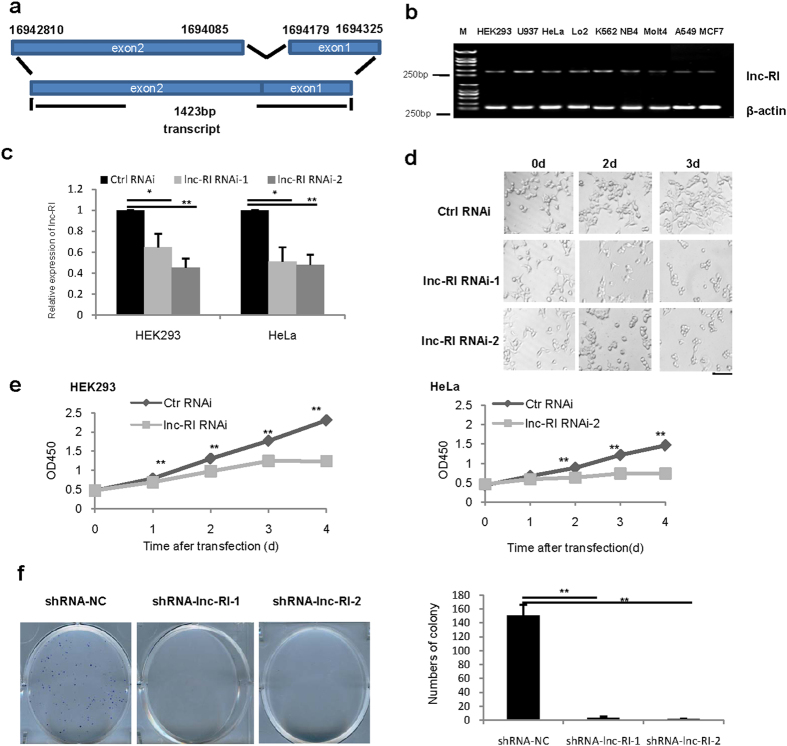
Effect of lnc-RI knockdown on cell growth. (**a**) Schematic of the lnc-RI locus. (**b**) Expression profile of lnc-RI in multiple cell types. (**c**) Effect of siRNAs on HEK293 cells and HeLa cells. HEK293 cells or HeLa cells were transfected with lnc-RI RNAi-1, lnc-RI RNAi-2, or Ctrl RNAi. Real time-PCR analysis was performed at 48 h after transfection. (**d**) Effect of the knockdown of lnc-RI on the morphology of HEK293 at 48 h post-transfection (Bar, 50 μm). (**e**) Effect of the knockdown of lnc-RI on growth of HEK293 and HeLa cells. HEK293 and HeLa cells were transfected with lnc-RI RNAi -2 and the numbers of cells were counted using Cell Counting Kit-8 at 1, 2, 3, and 4 d after transfection. (**f**) Effect of the knockdown of lnc-RI on colony formation of HeLa cells. HeLa cells were transfected with plasmid expressing lnc-RI shRNA. Cells were cultured with G418 (1000 ng/mL) for 14 days and numbers of colony were analysed. *P-value < 0.05, **P-value < 0.01.

Lnc-RI was recently identified by our group as an ionizing radiation-inducible transcript of a long non-coding RNA (lncRNA) with1423 nucleotides encoded by a gene located in chromosome 7p22.3 (ENST00000382528) and containing 2 exons (Fig. 1a). Although transcription of lnc-RI is significantly upregulated in response to ionizing radiation (Supplementary Fig. S1), its biological function was unknown. We examined the expression of lnc-RI in multiple human cell lines (including cancer cells and immortalized cells) using reverse transcription–PCR (RT-PCR) and found that it was ubiquitously expressed in all analyzed cell lines which indicated that expression of lnc-RI is not associated with cell type (Fig. 1b). To study the effects of lnc-RI on cellular function, two specific siRNA oligo nucleotides against lnc-RI were selected and transfected into HEK293 cells and HeLa cells. The knockdown results indicated that both lnc-RI siRNAs largely depleted the steady-state levels of lnc-RI in both cells (Fig. 1c). At the same time, we observed that numerous cells became rounded and died following the depletion of lnc-RI (Fig. 1d), which indicated that lnc-RI may be involved in cell growth or cell cycle regulation.

To investigate the effect of lnc-RI depletion on cell growth, HEK293 and HeLa cells were transfected with lnc-RI RNAi-2 or a scrambled control sequence, Ctrl RNAi. Cell growth curves were monitored after transfection. The growth curves indicated that the knockdown of lnc-RI significantly inhibited the proliferation of both cell lines (Fig. 1e). To further define the role of lnc-RI on cell proliferation, plasmids expressing shRNA targeted to lnc-RI (shRNA-lnc-RI-1 and shRNA-lnc-RI-2) were transfected into HeLa cells. The effectiveness of these shRNA expression vectors for silencing lnc-RI expression is shown in Supplementary Fig. S2. As shown in Fig. 1f, both shRNA-lnc-RI-1 and shRNA-lnc-RI-2 significantly inhibited colony formation of HeLa cells as compared with the control. Taken together, these data demonstrate that lnc-RI is essential for cell growth and survival.

### Depletion of lnc-RI disturbs mitotic progression and causes abnormal spindle construction

**Figure 2:**
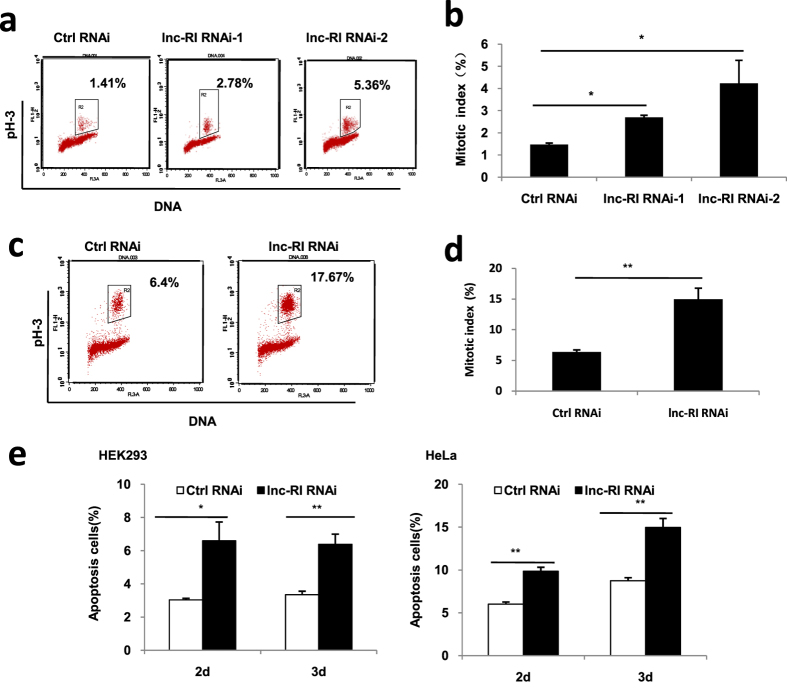
Effect of the knockdown of lnc-RI on cell mitosis and apoptosis. (**a,b**) Effect of knockdown on mitotic index of HeLa cells. HeLa cells were transfected with lnc-RI RNAi-1, lnc-RI RNAi-2, and Ctrl RNAi. At 48 h post-transfection, the cells were stained with PI and anti-p-H3 antibody and analysed by FACS. (**c,d**) Effect of the knockdown of lnc-RI on mitotic index of HEK293 cells 48 h after transfection. (**e**) Effect of the knockdown of lnc-RI on the apoptosis of HEK293 and HeLa cells analysed using Annexin-V-FITC apoptosis detection kit at 2 and 3 days after transfection. *P-value < 0.05,** P-value < 0.01.

In order to understand the critical role of Lnc-RI in cellular proliferation, the effect of lnc-RI on mitotic progression was analysed. HeLa cells were transfected with lnc-RI RNAi-1, lnc-RI RNAi-2, or Ctrl RNAi respectively. The cells were fixed and labelled with the antibody against p-H3, a marker of mitotic cells, and stained with PI at 48 h post-transfection. As shown in Fig. 2a,b, the percentage of mitotic cells after transfection with lnc-RI RNAi-1 or RNAi-2 was much higher than those in control cells. Similar results were also observed in HEK293 cells (Fig. 2c,d). In addition, the percentage of apoptotic cells was also increased in both HEK293 and HeLa cells at 2 and 3 days after transfection with lnc-RI RNAi-2 (Fig. 2e).

**Figure 3:**
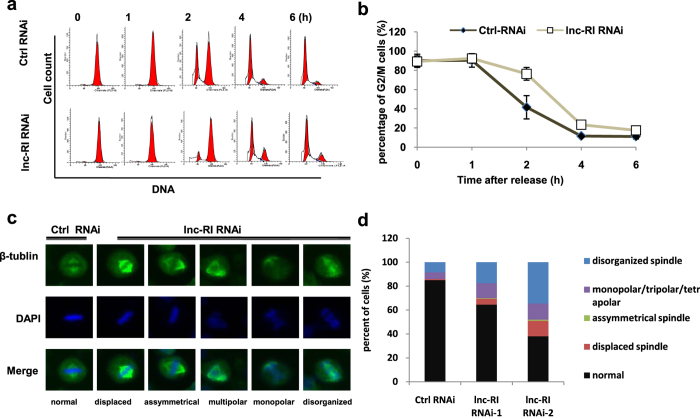
Knockdown of lnc-RI delays mitotic progression and induces aberrant spindles. (**a,b**) Knockdown of lnc-RI delayed M-phase progression. HeLa cells were transfected with lnc-RI RNAi-2 or Ctrl RNAi. At 12 h after transfection, the cells were treated with nocodazole (100 ng/mL) for 16 h. Mitotic cells were harvested using the mitotic shake-off method. Cell cycle profile was performed at the indicated time points after the cells were released. (**c**) The knockdown of lnc-RI induced aberrant mitotic spindle in HeLa cells. HeLa cells were transfected with lnc-RI RNAi-1, lnc-RI RNAi-2, and Ctrl RNAi and stained with anti-β-tubulin antibody and DAPI at 48 h after transfection. (**d**) The percentages of HeLa cells harbouring aberrant spindles were calculated in 200 randomly selected mitotic cells 48 h after siRNA transfection.

To further investigate the effects of knockdown of lnc-RI on mitosis, HeLa cells (HeLa cells is suitable for mitosis study) transfected with lnc-RI RNAi-2 or Ctrl RNAi were treated with nocodazole for 16 h. Synchronized mitotic cells were obtained using a shake-off method and were released after switching to nocodazole-free fresh medium. Cell cycle profiles were examined at various time points after release. As shown in Fig. 3a,b, 41.58% of the Ctrl RNAi-transfected cells remained in mitosis at 2 h after release. By contrast, 76.35% of the lnc-RI RNAi-2 transfected cells remained in mitosis, suggesting that there was a significant delay of mitotic exit with lnc-RI depletion.

The formation of a symmetrical bipolar spindle is one of the most important events in mitosis. Aberrant spindle formation could disturb mitotic progression, and induces mitotic arrest and genetic instability. To investigate the role of lnc-RI in regulating spindle formation, HeLa cells transfected with lnc-RI RNAi-1, lnc-RI RNAi-2, or Ctrl RNAi were stained with anti-β-tubulin antibody and DAPI at 48 h after transfection. Aberrant spindles were induced by transfection with lnc-RI RNAi and exhibited multiple morphological characteristics including asymmetrical spindle, displaced spindle, tripolar spindle, monopolar spindle, and disorganized spindle (Fig. 3c). A total of 200 mitotic cells were selected randomly from lnc-RI knockdown cells or control cells and were scored for aberrant spindles. The number of cells with aberrant spindles in lnc-RI knockdown cells was significantly higher than that of control cells (Fig. 3d). Taken together, these results demonstrate that lnc-RI plays a key role in proper spindle formation and mitotic progression.

### PLK1 is a key downstream mediator of lnc-RI to control mitosis

**Figure 4:**
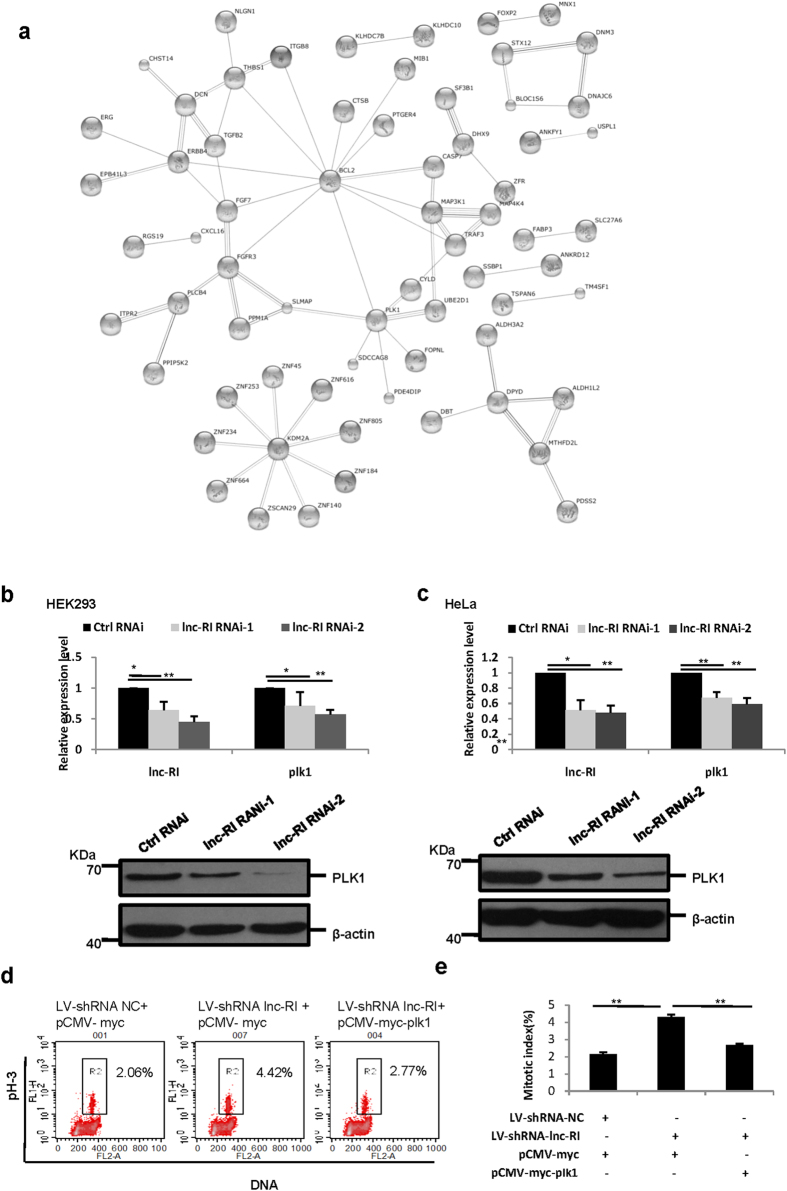
PLK1 is the target of lnc-RI and is involved in mitotic arrest induced by lnc-RI knockdown. (**a**) The functional association of lnc-RI target genes. All 144 candidate target genes were uploaded into STRING 10 (http://string-db.org/), and three networks related to cell cycle, apoptosis, and DNA damage were identified. (**b,c**) Knockdown of lnc-RI reduced PLK1 mRNA and protein expression levels. The HEK293 (**b**) and HeLa cells (**c**) were transfected with lnc-RI RNAi-1, RNAi-2 or Ctrl RNAi. At 48 h post-transfection, total RNA and total protein were extracted. Real-time PCR and western blot were performed to detect the expression of PLK1mRNA (upper) and protein (lower) after lnc-RI knockdown. (**d,e**) PLK1 expression rescued mitotic arrest induced by lnc-RI knockdown. 3 day after infection with LV-shRNA-lnc-RI, HeLa cells were transfected with plasmid expressing PLK1-myc and mitotic index was analyzed 48 h after transfection. *P-value < 0.05,**P-value < 0.01.

To explain the mechanism underlying the effects of lnc-RI in regulating spindle formation and mitotic progression, we screened for downstream genes regulated by lnc-RI. HeLa cells were transfected with lnc-RI RNAi-1, lnc-RI RNAi-2 or Ctrl RNAi, and alterations in gene expression were monitored using a Human Genome U133 Plus 2.0 expression profile cDNA microarray. The expression profile analysis identified 153 genes with altered expression, including 118 genes that were up-regulated greater than 1.5-fold in both lnc-RI RNAi-1 and lnc-RI RNAi-2-transfected cells, compared with Ctrl RNAi-transfected cells, and 35 genes that were downregulated (Supplementary Table S2). Search tool for the retrieval of interacting genes/proteins (http://string-db.org/)(STRING), a database of protein interactions, was used to investigate functional associations among the target genes of lnc-RI and networks involved in the biological function of lnc-RI. All of the genes with altered expression were uploaded into STRING 10 (except 8 genes not present in the database), which identified 3 gene networks. As shown in Fig. 4a, the 3 genes networks were involved in cell cycle (PLK1), apoptosis (BCL2), and the DNA damage response (KDM2A).

Among the three gene networks, we were most interested in PLK1, a well-known key regulator of mitosis from mitotic entry to exit. The cellular phenotypes by depleting lnc-RI observed in this study was very similar to those induced by PLK1 suppression as reported previously^13^,^14^,^15^,^16^. Next, we examined the association between PLK1 and lnc-RI. HEK293 cells and HeLa cells were transfected with lnc-RI RNAi-1, lnc-RI RNAi-2, or Ctrl RNAi, and PLK1 mRNA and protein expression levels were measured 48 h after transfection. As shown in Fig. 4b,c, both lnc-RI RNAi-1 and lnc-RI RNAi-2 reduced the expression of PLK1 mRNA and protein in both HEK293 and HeLa cells. As expected, mitotic arrest was observed in both HEK293 and HeLa cells transfected with PLK1 RNAi, which is similar to the effect induced by knocking down lnc-RI (Supplementary Fig. S3). We then examined whether PLK1 is a mediator of mitotic arrest induced by lnc-RI knockdown. HeLa cells were infected with LV-shRNA-lnc-RI according to the method above. 3 days after infection, expression of both lnc-RI and PLK1 were largely suppressed compared to the control cells (Supplementary Fig. S4). Plasmid expressing PLK1-myc fusion protein was transfected into HeLa cells at 3 days after infection with LV-shRNA-lnc-RI. Cells were harvested and the mitotic index was analysed 48 h after transfection. As shown in Fig. 4d,e and Supplementary Fig. S5, PLK1-myc rescued the mitotic arrest induced by knockdown of lnc-RI.

### Lnc-RI promotes PLK1 mRNA stability by competing miRNA-210-3p

**Figure 5:**
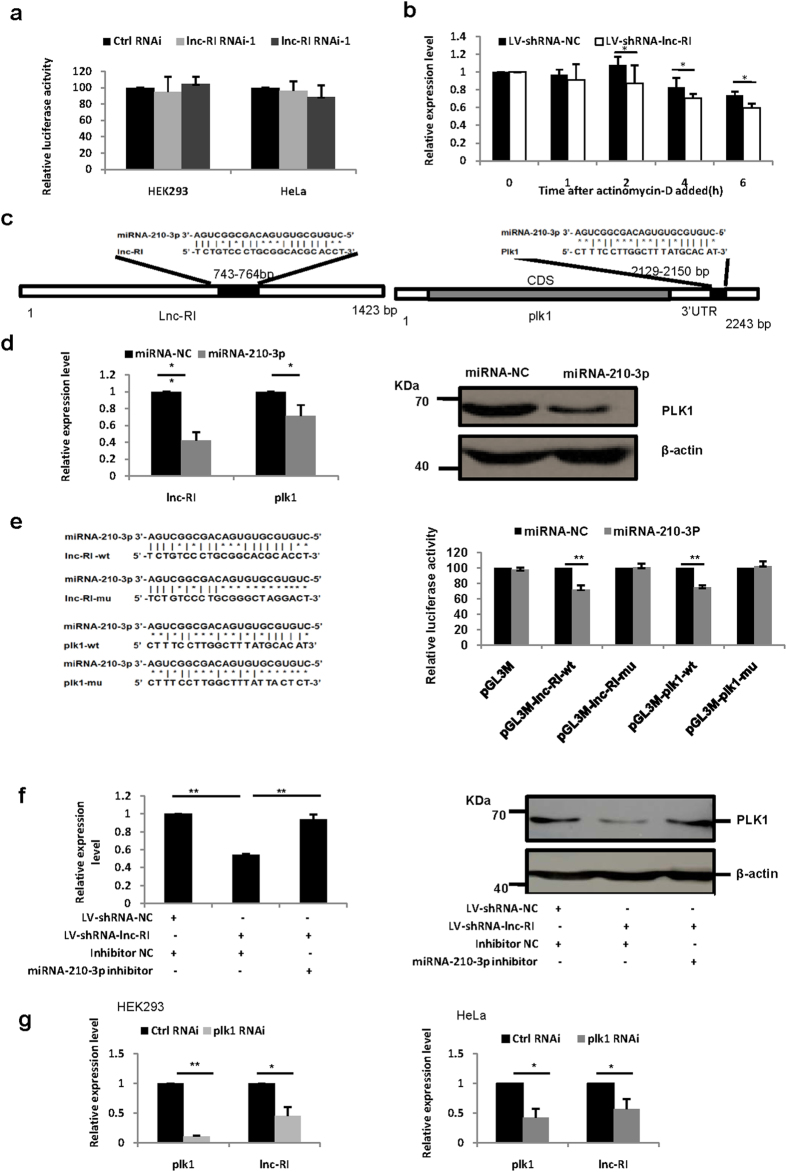
lnc-RI regulates PLK1 by competing with PLK1 mRNA for binding to miRNA-210-3p. (**a**) Effect of lnc-RI knockdown on PLK1 promoter activity. pGL3-PLK1 reporter plasmid containing the full length PLK1 promoter was co-transfected with lnc-RI RNAi-1, lnc-RI RNAi-2, or Ctrl RNAi in HeLa cells. Cells were harvested and luciferase activity was measured using a Dual Reporter assay system 48 h following transfection. (**b**) Effect of lnc-RI knockdown on PLK1 mRNA degradation. 3 day after infection with LV-shRNA-lnc-RI, HeLa cells were treated with actinomycin-D (5 μg/mL). Cells were harvested at 0, 1, 2, 4, and 6 h after actinomycin-D was added and PLK1 mRNA expression was analysed using Real-Time PCR. (**c**) Predicted sites of miRNA-210-3p on lnc-RI or PLK1 3′UTR. (**d**) Effect of miRNA-210-3p on lnc-RI and PLK1 in HeLa cells. HeLa cells were transfected with miRNA-210-3p mimics or Negative Control. At 48 h post-transfection, lnc-RI and PLK1 expression was measured using Real-Time PCR and western blot. (**e**) MiRNA-210-3p directly targets expression of lnc-RI and PLK1. Luciferase reporter vectors were generated by inserting the wild-type or mutated lnc-RI (674–810 bp) or 3′UTR of PLK1 (229–304 bp) into pGL3M plasmid. The reporter vectors were then co-transfected into HeLa cells with either miR-210-3p or NC. Cells were harvested for luciferase activity assay. (**f**) MiRNA-210-3p inhibitor rescued expression of PLK1. 3 day after infection with LV-shRNA-lnc-RI, HeLa cells were transfected with miRNA-210-3p inhibitor (100 nM) or control. At 24 h following transfection, cells were collected and PLK1 mRNA (left) and protein (right) expression were analyzed. (**g**) Knockdown of PLK1 reduced lnc-RI expression. HEK293 and HeLa cells were transfected with PLK1 siRNA. At 48 h after transfection, expression levels of lnc-RI and PLK1 were analysed using Real Time PCR. *P-value < 0.05, **P-value < 0.01.

To investigate the mechanism of lnc-RI to regulate the PLK1 expression, we first examined the effect of lnc-RI knockdown on the activity of the PLK1 promoter. As shown in Fig. 5a, knockdown of lnc-RI does not affect the activity of the PLK1 promoter in either HEK293 or HeLa cells. Next, we examined the effect of lnc-RI on the stability of PLK1 mRNA using HeLa cell infected with LV-shRNA-lnc-RI by inhibiting transcription using actinomycin-D. The results indicated that knockdown of lnc-RI promoted the degradation of PLK1 mRNA (Fig. 5b).

One of the major mechanisms of miRNA regulation of gene expression is to bind to the 3′UTR of target mRNA and promote mRNA degradation. It was reported that lncRNAs may affect mRNA stability by competing for binding to miRNAs^6^. To test the hypothesis that lnc-RI regulates PLK1 by this mechanism, we searched for a miRNA molecule which could target both the PLK1 mRNA and lnc-RI. A predicted targeting site of miRNA-210-3p was found in lnc-RI within the 743–764 bp region and in the PLK1 mRNA 3′UTR (Fig. 5c). Coincidently, He J. *et al*.^25^ reported that miRNA-210-3p could bind to 3′UTR of PLK1 mRNA and promote PLK1 mRNA degradation, and consequently induce aberrant spindles and mitotic arrest. Here, we have examined whether lnc-RI is another target of miRNA-210-3p. HeLa cells were transfected with miRNA-210-3p mimics. Expression of lnc-RI and PLK1 were measured 48 h after transfection. These results indicated that miRNA-210-3p reduced both lnc-RI and PLK1 expression (Fig. 5d). Next, we examined whether miRNA-210-3p regulates the expression of lnc-RI via binding to the 743–764 bp region of lnc-RI. The sequences of lnc-RI 674–810 bp or PLK1 3′UTR 229–304 bp containing miRNA-210-3p binding site were cloned separately downstream of the luciferase gene of the pGL3M plasmid, and co-transfected the plasmid with miRNA-210-3p into cells. These results showed that miR-210-3p significantly suppressed the luciferase activity (Fig. 5e) of both candidate target sequences. To further confirm the direct interaction between miRNA-210-3p and lnc-RI or PLK1, the binding sites in lnc-RI and PLK1 3′UTR were mutated. In comparison with wild-type plasmid, the binding site mutants rescued the repressive effects of miRNA-210-3p on luciferase activity (Fig. 5e). These results demonstrated that miRNA-210-3p regulates the expression of lnc-RI by directly binding to sites in lnc-RI and PLK1 mRNA.

To further examine whether lnc-RI regulates PLK1 expression by competing for miRNA-210-3p, HeLa infected with LV-shRNA-lnc-RI was transfected with miRNA-210-3p inhibitor and PLK1 mRNA and protein expression were analysed 24 h after transfection. As shown in Fig. 5e, miRNA-210-3p inhibitor rescued PLK1 reduction induced by knockdown of lnc-RI in HeLa cells. Next, we examined the effect of PLK1 knockdown on the expression of lnc-RI. As shown in Fig. 5g, knockdown of PLK1 reduced the expression of lnc-RI in both HEK293 and HeLa cells. Combining these results, we concluded that lnc-RI regulates PLK1 mRNA stability via competing for binding to miRNA-210-3p.

**Figure 6:**
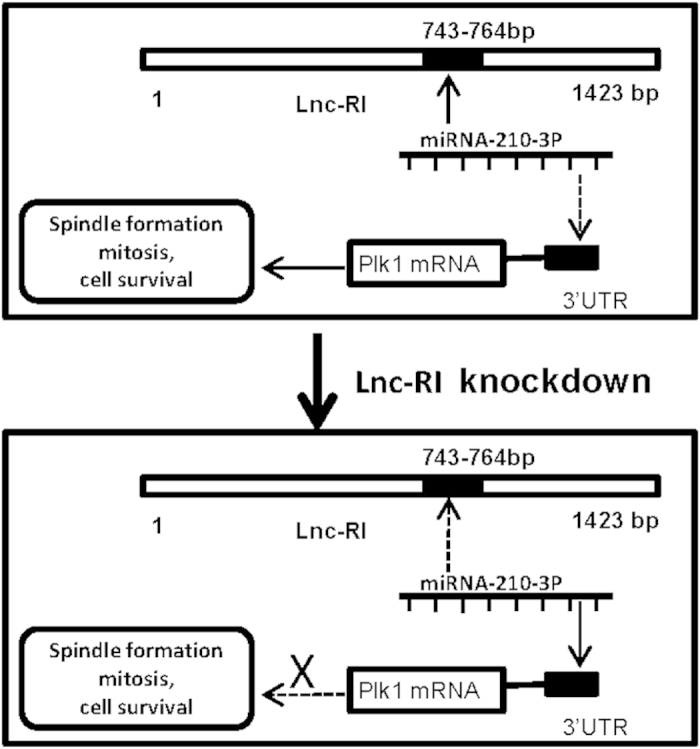
Schematic representation of the circuitry linking lnc-RI, miRNA-210-3p and plk1.

As described in Fig. 6, present study indicated that knockdown of lnc-RI induced aberrant spindle, mitotic arrest and apoptosis via down-regulating PLK1 mRNA stability by competing with the PLK1 mRNA 3′UTR for binding to miRNA-210-3p.

## Discussion

Increasing evidence indicate that lncRNAs play critical roles in multiple processes. Thus, lncRNAs are attracting much interest. To our knowledge, this study is the first report of clear evidence demonstrating the biological function of the newly identified long noncoding RNA, lnc-RI. In the present study, cell proliferation and colony formation assays demonstrated that lnc-RI is necessary for cell survival. Knockdown of lnc-RI induced aberrant spindles, mitotic arrest, and apoptosis, and mitotic regulator PLK1 was a mediator of knockdown of lnc-RI. Lastly, we found lnc-RI is a target of miRNA-210-3p and lnc-RI regulates PLK1 mRNA stability via competing for binding to miRNA-210-3p.

Spindle formation is a critical event during mitosis, and aberrant spindles activate mitotic arrest. Spindle formation is controlled by multiple regulators involved in centrosome formation, microtubule regulation, and chromosome segregation. Only one lncRNA has previously been found to be involved in spindle formation. Rošić S. *et al*. found that a long noncoding RNA named SAT III RNA binds to the kinetochore component CENP-C and is required for correct localization of the centromere-defining proteins CENP-A and CENP-C, as well as outer kinetochore proteins^26^. In this study, we identify lnc-RI as a novel regulator of spindle formation. Our data indicated that knockdown of lnc-RI caused spindle defects and consequent mitotic arrest and apoptosis. The genes altered induced by lnc-RI knockdown were examined, and 3 gene networks involved in cell cycle, cell apoptosis, and DNA damage were identified. Of the genes altered involved in the cell cycle, PLK1 has been shown to be a critical regulator of centrosome formation and plays an important role in mitosis and proper bipolar spindle formation^12^,^13^,^14^,^15^,^16^. In the present study, we found lnc-RI knockdown reduced PLK1 expression and PLK1 was indeed involved in mitotic arrest induced by lnc-RI knockdown.

LncRNAs regulate targets by different modes, including as competing endogenous RNA (ceRNA)^6^. According to the ceRNA hypothesis, lncRNAs may act as endogenous decoys for miRNAs, in turn affecting the binding of miRNAs to their mRNA targets. In this study, knockdown of lnc-RI promoted PLK1 mRNA degradation, so we hypothesized that lnc-RI regulates PLK1 as a ceRNA. To examine whether lnc-RI regulates PLK1 mRNA stability as a ceRNA, we searched for a miRNA which could bind simultaneously to lnc-RI and PLK1 mRNA 3′UTR. A predicted recognition site for miRNA-210-3p was found in the lncRNA743–764 bp region and miRNA-210-3p has been reported to bind to PLK1 mRNA 3′UTR^18^. Lnc-RI and PLK1 were validated as targets for miRNA-210-3p in our cellular models (Fig. 5b,c). Consistent with lnc-RI competing miRNA-210-3p with PLK1, we found that while depression of lnc-RI could reduce expression of PLK1, knockdown of PLK1 also reduced the levels of lnc-RI (Figs 4a and 5d). Lastly, our studies found miRNA-210-3p inhibitor could rescue the knockdown of PLK1 in lnc-RI knockdown cells, which supports the ceRNA regulatory mechanism of PLK1 by lnc-RI (Fig. 5f). He J. *et al*. reported that miR-210-3p could regulate PLK1 by binding PLK1 mRNA 3′UTR and thus disturb spindle formation and mitotic progress, which are similar to the phenotypes found in the present study.

In summary, this is the first study to demonstrate the function of lnc-RI. The data in this study indicate that lnc-RI is a novel regulator of mitosis via competing for miR-210-3p as a ceRNA to release PLK1 activity, thus playing a key role in proper spindle formation and mitotic progression.

## Methods

### Cell culture

HeLa and HEK293 cells (Kept in our lab) were cultured in PRIM-1640(Sigma) or DMEM(Gibco) containing 10% fetal bovine serum (Hyclone), 100 units/mL penicillin, and 100 μg/mL streptomycin in a humidified incubator at 37 °C in 5% CO2.

### MiRNAs, siRNAs, plasmids, and transfection

MiRNA mimics, miRNA inhibitors, and small interfering RNA (siRNA) duplexes were synthesized and purified by Shanghai GenePharma Co. (Shanghai, China). The plasmids expressing shRNAs targeting lnc-RI were constructed by Shanghai GenePharma Co. The sequences of miRNAs, miRNA inhibitors, siRNAs, and shRNAs are listed in Supplementary Table S1. Cells were transfected with siRNA, miRNA duplexes, or plasmids using Lipofectaime 2000 (Invitrogen Corp., Carlsbad, CA, USA) according to the manufacturer’s instructions.

### Cell Synchronization

To synchronize ce lls in the cell cycle, cells were treated with nocodazole (100 ng/mL) for 16 h. Floating, mitotic cells were then harvested by a shake-off method, washed twice, and reseeded into fresh medium.

### Antibodies

The following antibodies were used: rabbit anti-phospho-histone H3 (S10) polyclonal antibody (A301-844A, Bethyl, USA); mouse anti-β-tubulin (D-10) monoclonal antibody (ZS-5274, ZSGB-BIO, China); mouse anti-PLK1 (F-8) monoclonal antibody (sc-17783, Santa Cruz, USA); mouse anti-β-actin monoclonal antibody (60008-1-lg, Proteintech, USA); HRP-conjugated anti-mouse IgG (074-1806, KPL, USA); FITC-conjugated anti-rabbit IgG (02-15-06, KPL,USA); and FITC-conjugated anti-mouse IgG (02-18-06, KPL,USA).

### Western blot

Cells were harvested and washed twice with ice-cold PBS. Cell pellets were lysed in lysis buffer [50 mM Tris-HCl (pH 7.5), 150 mM NaCl, 1% (v/v) Nonidet P40, 0.5% sodium deoxycholate] with protease inhibitors. Cell lysates were subjected to SDS-PAGE and transferred to nitrocellulose membranes for western blot analysis.

### RT-PCR and Real-time PCR

Total RNA was extracted using RNA TRIzol (Invitrogen, US) according to the manufacturer’s instructions. PrimeScript RT reagent kit (Takara, Japan) was used to synthesize cDNAaccording to the manufacturer’s instructions. RT-PCR experiments were performed using PCR buffer (BioMed, China). Real-time PCR experiments were performed by the Taqman probe method. The primers and probes for PCR are listed in Table S1.

### Dual luciferase reporter assay

Cells were placed in 24-well plates 1d before transfection. On the next day, 100 ng of reporter plasmids and 1 ng of pRL-CMV internal control plasmid were co-transfected with siRNAs or miRNA mimics into HeLa cells. Cells were harvested 48 h after transfection. Luciferase activity was measured using a Dual Reporter assay system (Promega, USA). Firefly luciferase activityof each reporter was normalized to Renilla luciferase activity.

### Cell proliferation and colony formation assays

Cultures with 30% confluency were seeded in a 96-well plate 24 h before transfection. The number of cells transfected with siRNA for lnc-RI or negative control was counted using Cell Counting Kit-8 (Dojindo, Japan) at 0, 1, 2, 3, and 4 d after transfection. For colony formation assays, plasmids expressing shRNA to lnc-RI (also containing the neomycin-resistancegene) were transfected into HeLa cells. One day after transfection, 500 cells were reseeded into 6-well plates and cultured with G418 (1000 ng/mL) for 14 days and the number of resulting colonies was analysed.

### Cell cycle and apoptosis assays

For cell cycle analysis, the cells were harvested and fixed with 75% ethanol. The fixed cells were washed with PBS twice and permeabilised with 0.25% Triton X-100 in PBS for 15 min at room temperature. Cell pellets were incubated with the primary antibody (anti-phospho-Histone H3-Ser10, 1:1000) at room temperature for 30 min and with the secondary antibody (goat anti-rabbit IgG-FITC, 1:200) in the dark for 30 min. The cells were re-suspended in PBS plus 1 μg/mL RNase A, incubated for 20 min at 37 °C and stained with 25 μg/mL propidium iodide (PI). Apoptosis assays were performed using an Annexin-V-FITC apoptosis detection kit (Key GEN, China) according to the manufacturer’s protocol. Cell cycle and apoptosis assays were performed using a flow cytometer (BD FACS LSRII, USA).

### Immunofluorescence

Cells were grown on polylysine-coated glass coverslips. The cells were then fixed in PBS containing 4% formaldehyde for 15 min and permeabilised in Triton buffer (0.5% Triton X-100 in PBS). The fixed cells were blocked with blocking buffer (2% bovine serum albumin in PBS) in a humidified chamber for 30 min at 37 °C. Staining was performed using an anti-β-tubulin antibody (1:100) overnight at 4 °C in a humidified chamber and washed twice in a blocking buffer. The cells were incubated with FITC-conjugated anti-mouse (1:400) secondary antibodies. DNAs were stained with DAPI in a mounting solution. Images were obtained using a Zeiss Observer A1 microscope equipped with a Zeiss camera and software at magnification of 40×.

### Identification of downstream genes of lnc-RI by cDNA microarray

HeLa cells were harvested 48 h after transfection with Ctrl RNAi, lnc-RI RNAi-1, or lnc-RI RNAi-2. Total RNA was extracted and Human Genome U133 Plus 2.0 expressing profile chips (Affymetrix,USA) were used to screen the targets of lnc-RI by Capitalbio Technology Corporation (Beijing, China).

### Lentivirus preparation and infection of HeLa cells

The lentivirus expressing shRNA-lnc-RI-2 (LV-shRNA-lnc-RI) or negative control (LV-NC) were purchased from GenePharma (Shanghai, China). HeLa cells were infected with lentivirus (20MOI) according to the GenePharma Recombinant Lentivirus Operation Manual. After infection, puromycin (2 μg/ml) was added and cultured for 3 days. Real Time PCR was employed to analyze the expression levels of lnc-RI.

### Statistical analysis

Experiments were performed with at least three replicates in each assay or three independent experiments. Data is presented as mean ± S.D. Statistical analysis was performed using Student’s t-test. Significance was set at a probability value less than 0.05.

## Additional Information

**How to cite this article**: Wang, Z.-D. *et al*. Long noncoding RNA lnc-RI is a new regulator of mitosis via targeting miRNA-210-3p to release PLK1 mRNA activity. *Sci. Rep.* **6**, 25385; doi: 10.1038/srep25385 (2016).

## Supplementary Material

Supplementary Information
