# Supplementary material for: Long noncoding RNA lnc-RI is a new regulator of mitosis via targeting miRNA-210-3p to release PLK1 mRNA activity
Source: Sci Rep. 2016 May 10;6:25385. doi: 10.1038/srep25385 (PMC4861912; doi:10.1038/srep25385)
Supplement: Supplementary Information [file srep25385-s1.pdf]

Long noncoding RNA lnc-RI is a new regulator of mitosis via targeting miRNA-210-3p to release PLK1 mRNA activity

Zhi-Dong Wang[1,*,#], Li-Ping Shen[1,#], Cheng Chang[1,2], Xue-Qing Zhang[1],Zhong-Min Chen[1], Lin Li[1], Hong Chen[1] and Ping-Kun Zhou[1,2,*]

[1]Department of Radiation Toxicology and Oncology， Beijing Key Laboratory for Radiobiology, Beijing Institute of Radiation Medicine, Beijing, 100850, P. R. China.[2] Institute for Environmental Medicine and Radiation Hygiene, College of Public Health, University of South China, Hengyang, Hunan Province, 421000, P.R. China. #: These two authors contributed equally to this work. Correspondence and requests for materials should be addressed to P-K Z (email: zhoupk@bmi.ac.cn) and Z-D W (email: wangzhidong1977@126.com)

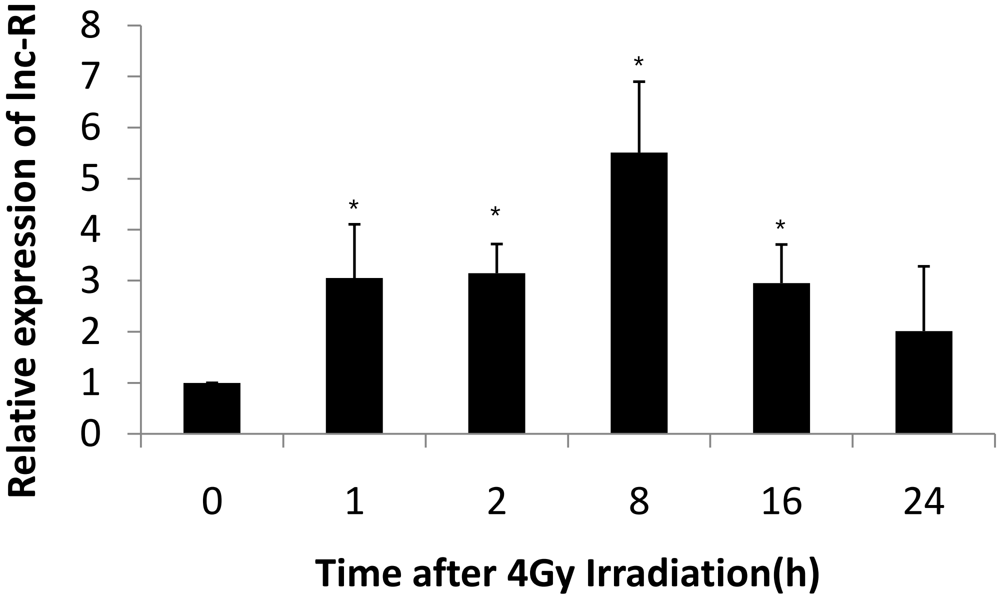

Supplementary Figure S1. Analysis of lnc-RI expression in HeLa cells exposed to 4Gy γ-rays. HeLa cells were expposed to to 4Gy γ-rays and cells were collected at different times point after radiation and total RNA was extracted and expression of lnc-RI were analyzed using Real Time PCR. * $p<0.05$.

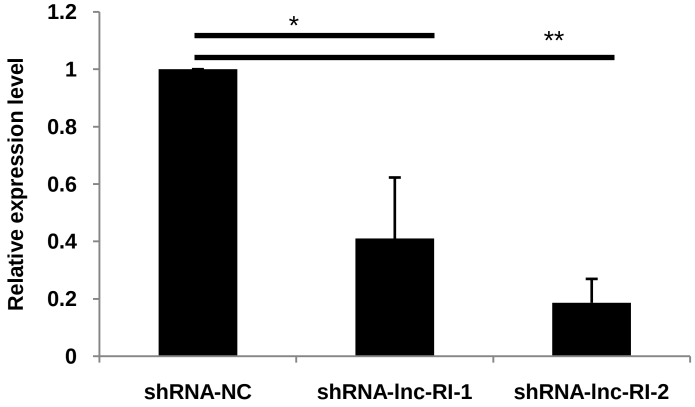

Supplementary Figure S2. Effect of shRNA-lnc-RI on expression of lnc-RI. HeLa cells were transfected with plasmids expressing shRNA targeting lnc-RI or control shRNA. 48h after transfection, lnc-RI epxression level were examine using Real Time PCR. * $p<0.05$;**<$p<0.01$

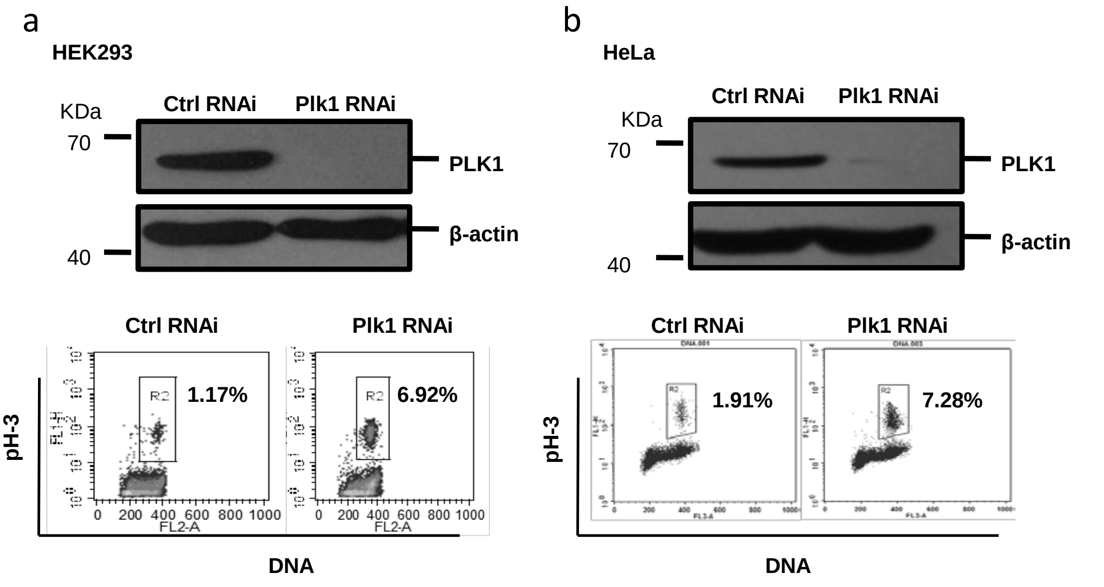

Supplementary Figure S3. Effect of plk1 knockdown on mitosis arrest in HEK293(a) and HeLa (b) cells. The HEK293 cells and HeLa cells were transfected with PLK1 RNAi or Ctrl RNAi. 48h after transfection, the cells were harvested and PLK1 expression (up) and mitotic index (down) were analysed.

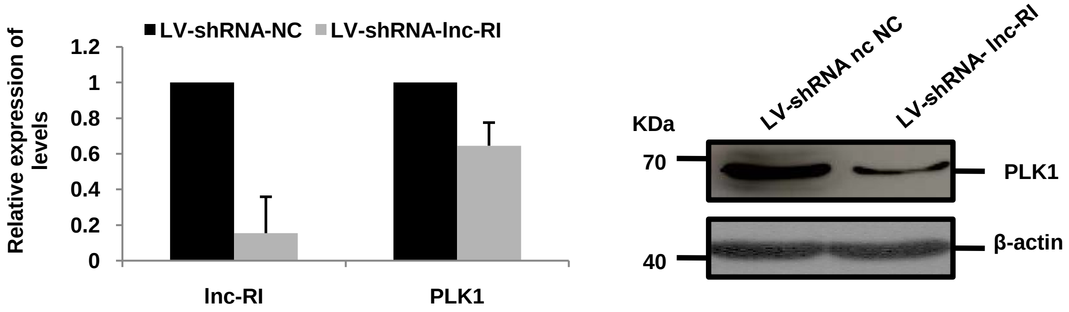

Supplementary Figure S4. Effect of LV-shRNA-lnc-RI on expression of lnc-RI and PLK1. HeLa cells were infected with LV-shRNA-lnc-RI or LV-shRNA-NC NA. 3d after infection, expression level lnc-RI and plk1 were examine using Real Time PCR and western blot.

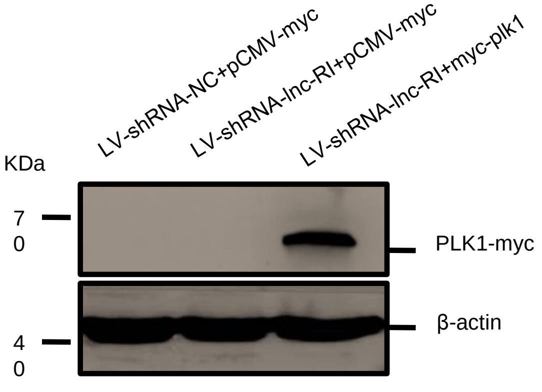

Supplementary Figure S5. Plk1-myc expression in HeLa infected with LV-shRNA-lnc-RI. HeLa cells were infencted with LV-shRNA-lnc-RI or LV-shRNA-NC. 3 days after infection ,cells were transfected with plasmid expressing plk1-myc or control plasmid and 48h after transfection, cells were harvested and plk1-myc expression was detected using myc tag antibody.

Table S1 Sequences of oligonucleotides

**siRNA sequence for lnc-RI or negative control**		
lnc-RI RNAi-1	Forward	5'GCCCAUGUCACCUAUGUGUTT3'
	Reverse	5'ACACAUAGGUGACAUGGGCTT3'
lnc-RI RNAi-2	Forward	5'GCUGAAUCCUCACAGCCUUTT3'
	Reverse	5'AAGGCUGUGAGGAUUCAGCTT3'
Ctrl RNAi	Forward	5'UUCUCCGAACGUGUCACGUTT3'
	Reverse	5'ACGUGACACGUUCGGAGAATT3'
plk1 RNAi	Forward	5'AGAUUGUGCCUAAGUCUCUTT3’
	Reverse	5'AGAGACUUAGGCACAAUCUTT3'
**Sequences of miRNA mimics**		
miRNA-NC	Forward	5'UUCUCCGAACGUGUCACGUTT3'
	Reverse	5'ACGUGACACGUUCGGAGAATT3'
hsa-miRNA-21(	Forward	5'CUGUGCGUGUGACAGCGGCUGA3'
	Reverse	5'AGCCGCUGUCACACGCACAGUU3'
**Sequences of miRNA inhibitor**		
miRNA inhibitor NC		5'CAGUACUUUUGUGUAGUACAA3'
hsa-miRNA-210-3p inhibitor		5'UCAGCCGCUGUCACACGCACAG3'
**Primers used for Real Time-PCR**		
actin	Forward	5'CCCTGGCACCCAGCAC3'
	Reverse	5'GCCGATCCACACGGAGTAC3'
	probe	5'ATCAAGATCATTGCTCCTCCTGAGCGC3'
lnc-RI	sense	5'CACACATCGTATACTCTGTCCCTG3'
	antisense	5'ACTGGTGAGCCTGAGTAGGTATG3'
	Probe	5'CACCAGGCAGTCTAAGCCCATGTCA3'
PLK1	Forward	5'GCAGCGTGCAGATCAACTTC3'
	Reverse	5'CCCGCTTCTCGTCGATGTAG3'
	probe	5'TCATCTTGTGCCCACTGATGGCAGC3'
**Primers used for RT-PCR**		
β-actin	Forward	5'CCTCTCCCAAGTCCACACAG3'
	Reverse	5'GGGCACGAAGGCTCATCATT3'
lnc-RI	Forward	5'ACCTGCTCTGGGGTTGCCAT3'
	Reverse	5'ACGTAGCCTTGCTGGGTGTG3'
**Sequences of shRNA target**		
shRNA-NC		5'GTTCTCCGAACGTGTCACGT3'
shRNA-lnc-RI-1		5'GCACATACCTACTCAGGCACA3'
ShRNA-lnc-RI-2		5'TGAATCCTCACAGCCTTGTTC3'

Table 2S List of genes changed after lnc-RI RNAi

Gene Symbol	Gene ID	Ratio of lnc-RNAi to Ctrl	
		lnc-RI RNAi-1	lnc-RI RNAi-2
PLCB4	AL535113	2.3926	3.3232
ZNF702P	NM_024924	2.295	3.0077
SLC27A6	NM_014031	2.0365	2.9896
LEPR	U50748	1.5217	2.871
ZNF616	BG170845	1.5554	2.8319
DCBLD2	AA722799	2.6239	2.7807
PREPL	BF445273	2.0768	2.7545
DDHD1	AA029818	1.6321	2.5603
UGT2A3	NM_024743	2.83	2.5137
ERBB4	AW772192	1.6757	2.3978
NEGR1	AI123532	1.5147	2.295
ENTPD4	AB002390	1.8359	2.2489
ABCA13	NM_152701	1.5738	2.2427
ELFN2	BC032083	1.8795	2.2182
DZIP1	NM_014934	1.895	2.2011
C7orf55	BF540829	1.5702	2.1669
CHD9	AW971892	1.7769	2.1619
DPYD	NM_000110	1.7498	2.1619
PLDN	BC004819	1.7694	2.1521
CTSB	AA020826	1.5633	2.1443
CYLD	AI453099	1.5322	2.1408
CPA3	NM_001870	1.7506	2.1333
NLGN1	AI912122	2.0819	2.122
PEX13	BC040953	1.9908	2.0933
MESDC2	AK026606	2.0005	2.0873
CHST14	AA039350	1.5835	2.0781
THBS1	NM_003246	1.5281	2.0652
HS2ST1	AW151887	1.6945	2.064
FGF7	AF523265	1.5977	2.0424
MNX1	AI738662	1.7579	2.0136
TBC1D9	AI348094	1.8478	2.0065
PLEKHB2	NM_017958	1.5175	1.9389
ZNF253	AV683221	1.6316	1.9324
LOC253264	AK023445	1.9305	1.9121
GFPT1	AI268315	1.5147	1.8984
FGFR3	NM_000142	1.5121	1.8829
GANAB	W87689	1.7003	1.8813
ZNF45	NM_003425	1.9453	1.8599
ARHGEF9	NM_015185	1.6156	1.8597
GK5	AI866260	1.5399	1.8562
PLA2G12A	NM_030821	1.5864	1.8557
SNX16	AW150236	2.606	1.8502
HABP4	AF241831	1.5861	1.8486
MTHFD2L	AA702016	1.9015	1.809
PREX1	BF308645	1.7175	1.8035

Gene Symbol	Gene ID	Ratio of lnc-RNAi to Ctrl	
		lnc-RI RNAi-1	lnc-RI RNAi-2
PTGER4	AA897516	1.6327	1.8033
PTGR2	BE465894	2.0819	1.7925
HSDL2	BC004331	1.5594	1.7919
STX12	AI816243	1.6652	1.7866
MAP4K4	NM_017792	1.5179	1.7808
KLHDC7B	AI631846	1.5374	1.778
ERG	AA296657	1.512	1.7746
CCDC88C	AB040942	1.5976	1.7657
PDSS2	NM_020381	1.7175	1.7593
CASP7	NM_001227	1.5214	1.7543
MIB1	BE048628	1.8546	1.7532
DNM3	AI631915	1.5253	1.751
G3BP2	AB014560	1.9389	1.7453
C2CD2	AP001745	2.0207	1.7406
STXBP5	AI825998	1.5373	1.7307
ITPR2	AA834576	1.5351	1.7181
FOXP2	AW291402	1.7798	1.7177
NTN4	AF278532	1.5724	1.7158
CD164	BF669455	2.0885	1.713
BCL2	NM_000657	1.7844	1.7043
TGFB2	M19154	1.6333	1.6988
CREBL2	AL529409	1.5973	1.6981
PDE4DIP	H15535	1.7811	1.696
KDM2A	BE675843	1.5576	1.6859
UBE2D1	AL545760	1.5717	1.685
MGC16385	NM_145039	1.557	1.6848
FAM13C	BC036453	1.8021	1.6713
FBXO45	AA973128	1.5357	1.6692
BDH2	AW136198	1.5214	1.6652
ZNF234	BE909177	1.6242	1.6573
DNAJC6	AV729634	1.5857	1.6568
LYPD6	AA227842	1.5659	1.6557
RP9P	AK091223	1.831	1.653
C16orf63	AA524072	1.6505	1.6519
TCF4	BF592782	1.8309	1.6499
SLC33A1	NM_004733	1.6151	1.6473
C9orf86	BE783949	1.8049	1.644
SPRED1	BE967019	1.5009	1.6413
COX11	AI376724	1.6334	1.6406
FARSB	AW024617	1.836	1.6361
ZSCAN29	BE622486	1.5754	1.6352
THAP5	AI878997	1.7907	1.6336
ALDH1L2	AI654224	1.6492	1.6266
ITGB8	BF513121	1.7746	1.625
USPL1	AL832776	1.5188	1.6224
PPM1A	AI241945	1.9581	1.6218

Gene Symbol	Gene ID	Ratio of lnc-RNAi to Ctrl	
		lnc-RI RNAi-1	lnc-RI RNAi-2
HISPPD1	NM_015216	1.556	1.6139
MAMDC2	AI862120	1.6479	1.6124
ATF1	CA337502	1.6167	1.6116
EPB41L3	AI770004	1.5316	1.6037
KLHDC10	D87454	1.7923	1.6007
ZNF664	BE965646	1.5616	1.5931
CCDC85A	AI376433	1.5337	1.5734
ANKRD12	AW572909	1.7447	1.5727
FAM173B	AI384017	1.5794	1.5687
LPP	BF221852	1.6944	1.5631
ZNF805	AV726376	1.7775	1.5527
PCDH7	BE644809	1.7188	1.5522
ZNF140	NM_003440	1.5526	1.5495
TMTC2	AI862551	1.6854	1.5481
TRAF3	AI721219	1.593	1.5472
DBT	M27093	1.6393	1.546
ZNF184	AI811577	1.5365	1.5365
FKBP7	AA683602	1.9764	1.5354
LOC339929	BC042414	1.6258	1.5317
DCN	AI336924	1.5271	1.5287
TPM4	BC002827	2.1417	1.5244
SDCCAG8	NM_006642	1.7163	1.5198
RGS19	NM_005873	1.7565	1.5167
SLMAP	BF526855	1.7083	1.5166
TTC3	BC026260	1.6782	1.5083
TMEM64	AA524005	0.5884	0.669
DHX9	BF313832	0.5595	0.6663
CABP4	BC033167	0.6478	0.6607
CXCL16	AF275260	0.5663	0.6518
ALDH3A2	BC002430	0.5319	0.6454
PRO2949	NM_018544	0.4912	0.6436
C14orf182	BF691523	0.6122	0.6373
DPEP1	NM_004413	0.5617	0.6335
PDLIM5	NM_006457	0.6511	0.6244
DKFZp686O1327	U80770	0.5434	0.6196
VMA21	BG036203	0.6202	0.6092
KRT17	NM_000422	0.6629	0.6055
ANKFY1	AK025960	0.6089	0.6027
PLK1	NM_005030	0.659	0.598
SSBP1	BC008402	0.6604	0.5876
SF3B1	AW003030	0.6645	0.586
FABP3	NM_004102	0.647	0.5802
MAP3K1	AA361361	0.6501	0.5738
FAM175A	AK021582	0.4781	0.5737
C3orf43	AL833443	0.6259	0.5723

Gene Symbol	Gene ID	Ratio of lnc-RNAi to Ctrl	
		lnc-RI RNAi-1	lnc-RI RNAi-2
LIG4	AI829314	0.5649	0.5387
TSPAN6	AF053453	0.646	0.5193
C1orf125	NM_144696	0.6089	0.5158
LTB	BC018898	0.5686	0.5129
GPR125	BC026009	0.6088	0.4713
LOC221710	AI870473	0.6511	0.4548
CPEB4	AV704132	0.559	0.4543
FAM111B	AA960844	0.6064	0.4329
ZFR	BF445199	0.644	0.4154
TM4SF1	AI760128	0.5781	0.4105
TANC2	NM_015623	0.4068	0.3502
CEACAM7	NM_006890	0.5827	0.3016
COL10A1	X98568	0.5416	0.3012
